# Intraoperative radiotherapy: An alternative to whole‐breast external beam radiotherapy in the management of highly selective breast cancer: A SEER database analysis

**DOI:** 10.1002/cam4.7458

**Published:** 2024-08-19

**Authors:** Dexun Sun, Guanhua Lu, Fenmei Liang, Wangjian Zhang, Tao Zeng, Yun Ling, Haojie Peng, Ting Xia, Meilin Hu, Xinxin Chen

**Affiliations:** ^1^ Department of Breast Surgery The Second Affiliated Hospital of Guangzhou Medical University Guangzhou Guangdong China; ^2^ Department of Medical Statistics, School of Public Health Sun Yat‐sen University Guangzhou Guangdong China

**Keywords:** breast cancer, intraoperative radiotherapy (IORT), SEER database, survival time, whole‐breast external beam radiotherapy (EBRT)

## Abstract

**Objective:**

This study aimed to verify if intraoperative radiotherapy (IORT) can achieve the same survival outcome as whole‐breast external beam radiotherapy (EBRT) in early breast cancer after breast‐conserving surgery (BCS), and to explore the suitable candidates that can safely receive IORT after BCS.

**Methods:**

Eligible post‐BCS patients who received IORT or EBRT were included in the Surveillance, Epidemiology and End Results (SEER) database from 2010 to 2018. Risk factors that affected 5‐year overall survival (OS) or breast cancer specific survival (BCSS) were identified by Cox proportional hazards regression analysis. Clinical characteristics, OS, and BCSS were comparatively analyzed between the two treatment modalities.

**Results:**

The survival analysis after propensity score matching confirmed that patients who received IORT (*n* = 2200) had a better 5‐year OS than those who received EBRT (*n* = 2200) (*p* = 0.015). However, the two groups did not differ significantly in 5‐year BCSS (*p* = 0.381). This feature persisted even after multivariate analyses that took into account numerous clinical characteristics. Although there was no significant difference in BCSS between different subgroups of patients treated with IORT or EBRT, patients over 55 years of age, with T1, N0, non‐triple negative breast cancers, hormone receptor‐positive, and histologic grade II showed a better OS after receiving IORT.

**Conclusion:**

In low‐risk, early‐stage breast cancer, IORT was not inferior to EBRT considering 5‐year BCSS and OS. Considering the equivalent clinical outcome but less radiotoxicity, IORT might be a reasonable alternative to EBRT in highly selective patients undergoing BCS.

## 1 INTRODUCTION

Whole‐breast external beam radiotherapy (EBRT) has been the international standard radiotherapy regimen after breast‐conserving surgery (BCS) for early‐stage breast cancer,^1^, ^2^ patients who received whole‐breast irradiation had a 15.7% lower 10‐year risk of recurrence and a 3.8% lower 15‐year risk of breast cancer death compared with patients who did not receive whole‐breast irradiation.^3^ The standard regimen for EBRT is 45–50 Gy in 25–28 fractions for 5–7 weeks of radiotherapy to the whole breast. To shorten the course of radiotherapy, some studies have used a hypofractionated regimen, which is 39–42.9 Gy in a single fractional dose of 2.6–3.3 Gy divided into 13 fractions to be radiographed within 5 weeks.^4^ However, EBRT is associated with postoperative complications, including breast deformity morbidities, superficial ulcers of the skin, and local sensory dysfunction.^5^, ^6^ In addition to the fact that hypofractionated regimens may be more likely to result in the adverse effects described above than conventional fractionation regimens, certain patients with other co‐morbidities or who are more frail may be less able to tolerate the effects of hypofractionated regimens. At the same time, postoperative radiotherapy‐related factors such as the long treatment duration, cost of treatment, and far distance to treatment centers also prevent some patients from receiving postoperative radiotherapy.^5^, ^7^, ^8^ What is more, in the era of effective multimodal therapy, full conventional EBRT irradiating the whole breast may now be unnecessary in all patients, especially those with a very low risk of recurrence. Consequently, new concepts of more personalized risk‐adapted approaches have been tested and applied in clinical settings.

As a de‐escalation postoperative radiotherapy, intraoperative radiotherapy (IORT)^9^ is a treatment strategy in which postoperative EBRT is substituted by a single shot of radiation to the tumor bed at an 18–24 Gy dose given during the surgical procedure.^10^ Results of randomized controlled clinical studies of IORT in early breast cancer suggest satisfactory outcomes in terms of overall survival and late adverse effects. Patients who undergo IORT alone have less post‐therapy morbidities than those who undergo EBRT.^11^, ^12^, ^13^, ^14^, ^15^, ^16^ These results made IORT seem to be a reasonable alternative to whole‐breast EBRT in specific patients. In the meantime, IORT as a one‐time therapy reduces treatment duration and noncompliance to postoperative radiation. However, electron intraoperative radiotherapy (ELIOT) trial^13^ reported a significantly higher risk of ipsilateral breast tumor recurrence in patients in the IORT group (4.4%) compared to patients in the EBRT group (0.4%), which means that we have to balance the tumor recurrence risk and convenience of IORT and select proper candidates for IORT. Perhaps only rigorously screened patients who are hormone receptor‐positive and human epidermal growth factor receptor 2 (HER2) negative, with small tumors and no axillary lymph node metastases, should be treated with IORT as an alternative to EBRT.

Considering EBRT may be unnecessary in all patients. Stricter selection of candidates with early breast cancer for IORT is mandatory to avoid unnecessary EBRT in these patients while ensuring their clinical efficacy. In this study, we selected low‐risk (T1–T2, N0–N1, M0) patients who underwent BCS, from the Surveillance, Epidemiology, and End Results (SEER) database, which is a large, nationwide population‐based registry. The survival outcomes of patients in the IORT group were compared to the patients in the EBRT group to find out whether the patients we selected were the proper candidates for IORT. We also evaluated clinicopathological factors significantly associated with clinical outcomes in the IORT and EBRT groups.

## 2 METHODS

### 2.1 Design, data source, and study cohort

We categorized early‐stage breast cancer patients undergoing BCS into IORT and EBRT groups according to whether they received only IORT or EBRT, and compared the 5‐year overall survival (OS) and breast cancer specific survival (BCSS) of the two groups.

The National Cancer Institute's SEER database, the authoritative source of information on cancer incidence and survival in the United States, is a comprehensive, publicly accessible cancer database covering about 48.0% of the US population. Our data were derived from the SEER*Stat software (version 8.4.0) provided by the National Cancer Institute, SEER Research Plus Data, 18 Registries, November 2020 Sub (2000–2018). SEER databases are public, retrospective, and therefore do not require informed consent. Because the SEER databases began tracking information on HER2 status in 2010, the start of this study began in 2010. The SEER database does not emphasize whether the EBRT performed by the patient was conventionally segmented or hyperegmented.

IORT techniques are as follows: (1) Beam radiation; (2) Combination of beam with implants or isotopes; (3) Radiation, NOS method or source not specified; (4) Radioactive implants (includes brachytherapy); (5) Radioisotopes.

We collected breast cancer patients who received BCS (Partial mastectomy/Partial mastectomy with nipple resection/Lumpectomy or excisional biopsy/Re‐excision of the biopsy site for gross or microscopic residual disease/Segmental mastectomy; Special site surgery code: 20–24) from 2010 to 2018. We enrolled only patients who received a single radiotherapy treatment. Exclusion criteria: (1) T≥T3, N≥N2, M≥M1; (2) Did not receive radiotherapy; (3) Patients with missing information; (4) The pathologic type was not ductal, lobular, or mixed (ductal and lobular); (5) Histologic grading ≥grade IV, multiple and bilateral masses; (6) Did not receive IORT or EBRT.

### 2.2 Study variables and outcomes

Demographic and disease characteristics identified and collected in the SEER database for this study include age, ethnicity, T stage, N stage, chemotherapy, systemic/surgery sequence, estrogen receptor (ER) status, progesterone (PR) status, HER2 status, marital status, patient's hospital of regions, histological grade, histological type. The primary endpoint was OS, and the second endpoint was BCSS, with OS defined as the duration from the day of diagnosis to death. BCSS is defined as the time from initial diagnosis to death from any cause related to cancer. Cohort patients were followed until patient death and ended in 2018.

### 2.3 Statistical analysis

Categorized data are presented as a number (*N*) or a percentage (%). All statistical analyses were performed using R language (version 4.2.0). To eliminate significant differences in baseline covariates and inherent selection bias, propensity score matching (PSM) analyses were performed between the IORT and EBRT groups. The PSM is a tool to narrow selection bias and achieve a balance of variables across treatment groups in non‐randomized studies. IORT and EBRT were matched in a 1:1 ratio using a propensity model. Clinicopathological features that have a significant impact on clinical outcomes: age, T stage, N stage, chemotherapy, systemic/surgery sequence, breast cancer type (triple negative or non‐triple negative), ER status, PR status, HER2 status, histological grade, and histological type. The Kaplan–Meier survival curve was used for survival analysis between the IORT group and the EBRT group. An univariate Cox regression survival test was used for hypothesis testing to determine the risk prognostic factors affecting OS and BCSS. Multivariate Cox regression was used to analyze the independent prognostic risk factors. Corresponding hazard ratios (HR) and 95% confidence intervals (CI) were reported.

There were no lost patients in this study because of the exclusion of patients with incomplete information.

## 3 RESULTS

### 3.1 Screening process

**FIGURE 1:**
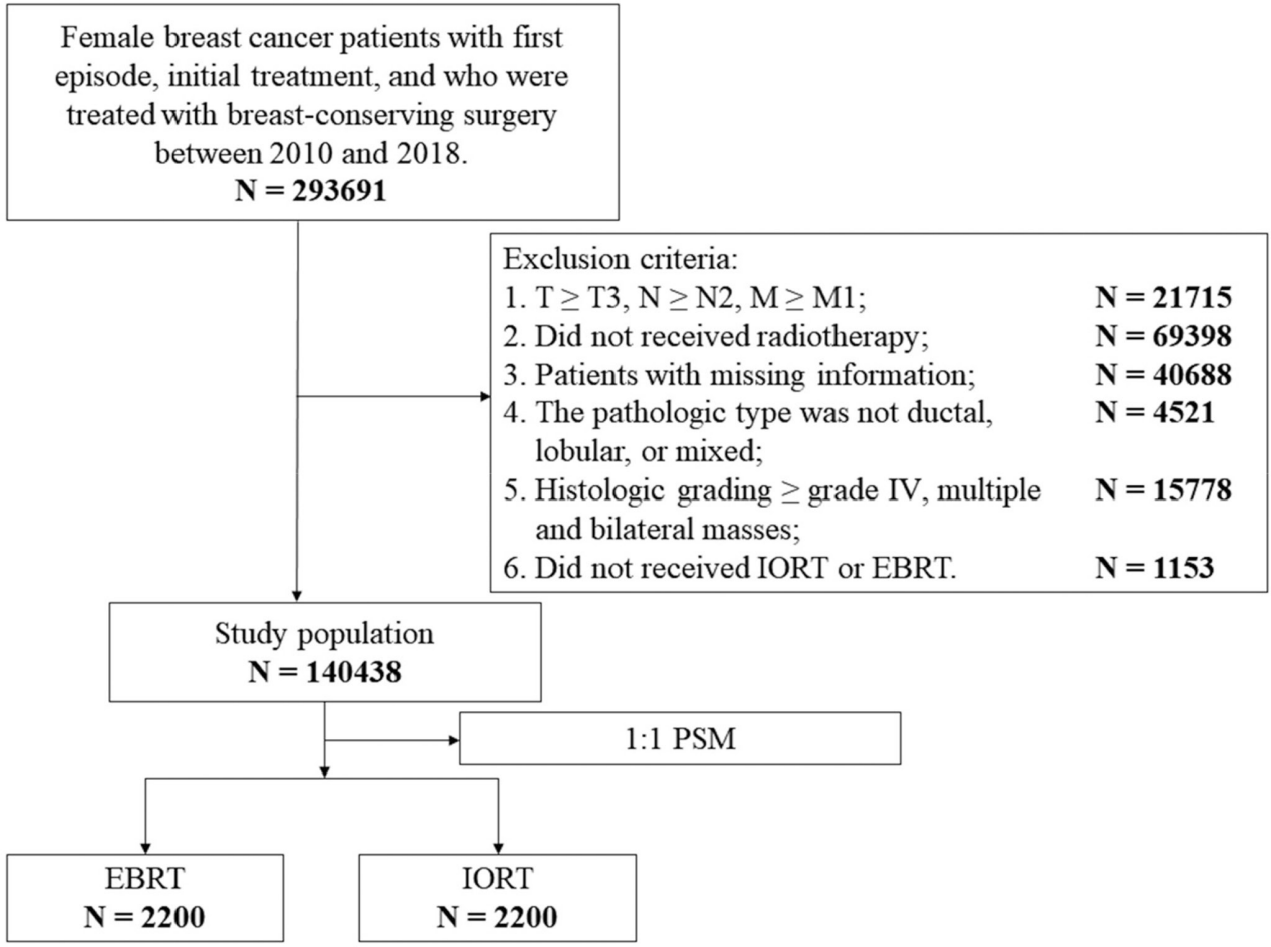
Flow chart of patient selection. IORT, intraoperative radiotherapy; EBRT, external beam radiotherapy; PSM, propensity score matching.

**FIGURE 2:**
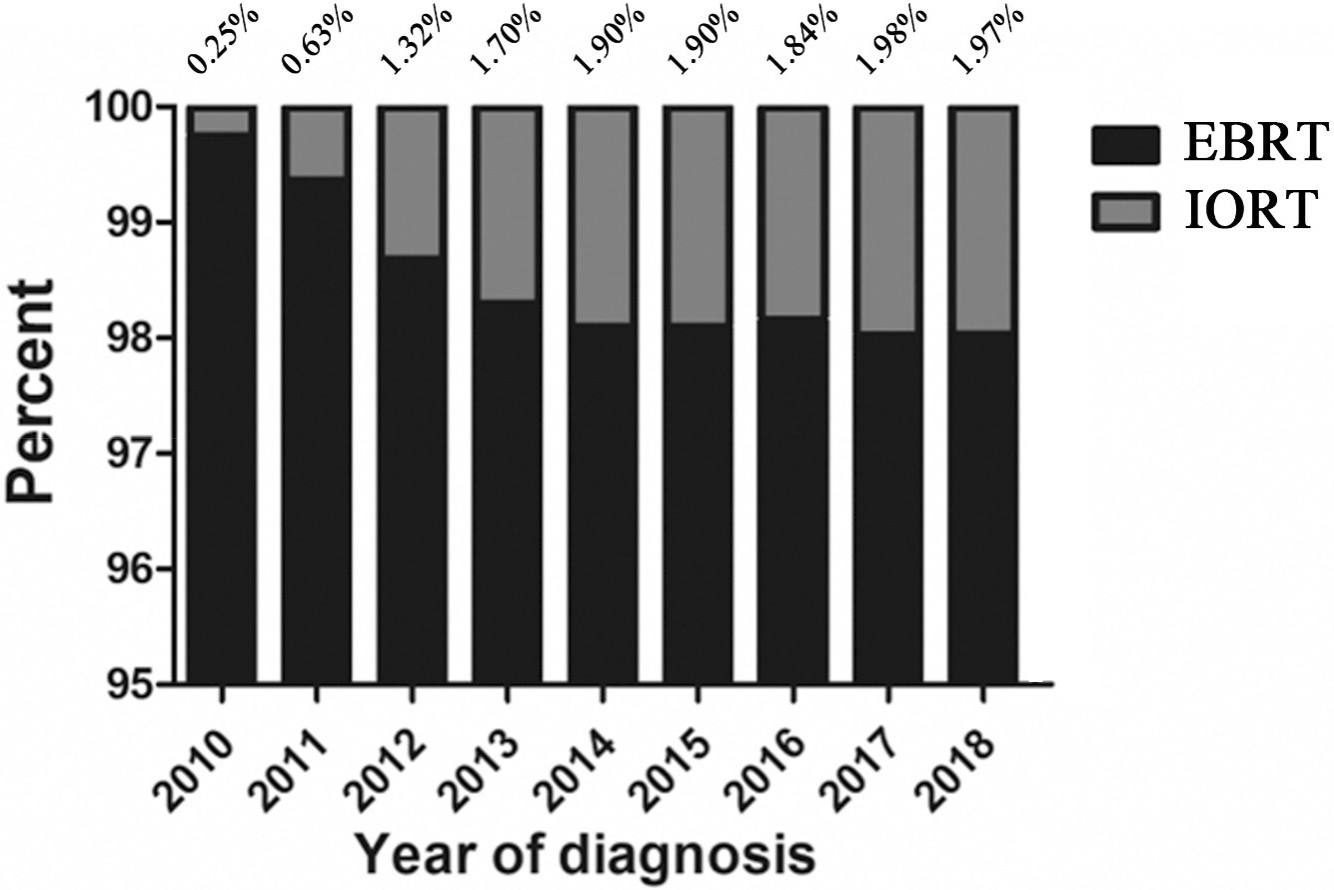
Percent of patients who received EBRT or IORT between 2010 and 2018. IORT, intraoperative radiotherapy; EBRT, external beam radiotherapy. The figure illustrates the ratio of IORT to the total number of radiotherapy patients in the year.

From 2010 to 2018, we collected a total of 293,691 female breast cancer patients undergoing BCS in the SEER database, and excluded 153,253 patients after exclusion screening, with a total of 140,438 eligible patients (Figure 1). According to the proportion of the IORT group and EBRT group per year, we made a proportion distribution map from 2010 to 2018 (Figure 2), which could be intuitively found that the proportion of patients in the IORT group increased year by year from 2010 to 2014 (0.25%–1.90%). The proportion of patients with IORT and EBRT remained relatively stable (1.90%–1.98%) from 2014 to 2018. A total of 4400 patients were enrolled in the study after a 1:1 PSM between 2200 patients receiving IORT and the EBRT group.

### 3.2 Baseline characteristics

Among the 4400 patients who met the analysis criteria, the important characteristics (age, T stage, N stage, chemotherapy, systemic/surgery sequence, breast cancer type (triple negative or non‐triple negative), ER status, PR status, HER2 status, histological grade, and histological type) of the two groups were well matched (Table 1). Eligible patients were early‐stage: 3639 (82.70%) were older than 55 years, 3835 (87.16%) were T1, 4209 (95.66%) were N0, 3963 (90.07%) did not receive chemotherapy The pathologic features of the majority of patients showed a low‐risk profile: 4270 (97.05%) were non‐triple negative breast cancer, 4217 (95.84%) were ER‐positive, 3879 (88.16%) were PR‐positive, and 4189 (95.20%) were HER2‐negative. Those with histologic classification of grade I and II (*n* = 3856, 87.64%) accounted for the largest number of patients. These clinical characteristics were not significantly different in the IORT and EBRT groups, which meant that they were comparable between the two groups.

**TABLE 1:** Baseline characteristics after propensity score matching.

Characteristic	Overall	IORT	EBRT	*p*‐value
	*N* = 4400	*N* = 2200	*N* = 2200	
Age group (%)				
≤55	761 (17.30)	380 (17.27)	381 (17.32)	1.000
>55	3639 (82.70)	1820 (82.73)	1819 (82.68)	
Ethnicity (%)				
Non‐Hispanic	4094 (93.05)	2030 (92.27)	2064 (93.82)	0.058
Hispanic	306 (6.95)	170 (7.73)	136 (6.18)	
T stage (%)				
T1	3835 (87.16)	1918 (87.18)	1917 (87.14)	1.000
T2	565 (12.84)	282 (12.82)	283 (12.86)	
N stage (%)				
N0	4209 (95.66)	2104 (95.64)	2105 (95.68)	1.000
N1	191 (4.34)	96 (4.36)	95 (4.32)	
Chemotherapy (%)				
No	3963 (90.07)	1982 (90.09)	1981 (90.05)	1.000
Yes	437 (9.93)	218 (9.91)	219 (9.95)	
Systemic/surgery sequence (%)				
Neoadjuvant therapy	42 (0.95)	21 (0.95)	21 (0.95)	0.987
Adjuvant therapy	3114 (70.77)	1558 (70.82)	1556 (70.73)	
Others	59 (1.34)	28 (1.27)	31 (1.41)	
No systemic therapy	1185 (26.93)	593 (26.95)	592 (26.91)	
Breast cancer type (%)				
Non‐triple negative	4270 (97.05)	2135 (97.05)	2135 (97.05)	1.000
Triple negative	130 (2.95)	65 (2.95)	65 (2.95)	
ER status (%)				
Negative	183 (4.16)	92 (4.18)	91 (4.14)	1.000
Positive	4217 (95.84)	2108 (95.82)	2109 (95.86)	
PR status (%)				
Negative	521 (11.84)	261 (11.86)	260 (11.82)	1.000
Positive	3879 (88.16)	1939 (88.14)	1940 (88.18)	
HER2 status (%)				
Negative	4189 (95.20)	2094 (95.18)	2095 (95.23)	1.000
Positive	211 (4.80)	106 (4.82)	105 (4.77)	
Marital status (%)				
Married/unmarried or domestic partner	2567 (58.34)	1305 (59.32)	1262 (57.36)	<0.001
Single (never married)	659 (14.98)	375 (17.05)	284 (12.91)	
Separated/divorced/widowed/unknown	1174 (26.68)	520 (23.64)	654 (29.73)	
Regions (%)				
Low SES	3964 (90.09)	2041 (92.77)	1923 (87.41)	<0.001
High SES	436 (9.91)	159 (7.23)	277 (12.59)	
Histological grade (%)				
Grade I	1745 (39.66)	873 (39.68)	872 (39.64)	0.996
Grade II	2111 (47.98)	1054 (47.91)	1057 (48.05)	
Grade III	544 (12.36)	273 (12.41)	271 (12.32)	
Histological type (%)				
IDC	3748 (85.18)	1873 (85.14)	1875 (85.23)	0.990
ILC	221 (5.02)	110 (5.00)	111 (5.05)	
Mixed	431 (9.80)	217 (9.86)	214 (9.73)	

*Note*: Categorized data are presented as a number (*N*) or a percentage (%). *p* ≤ 0.05 indicates statistical significance.

Abbreviations: EBRT, external beam radiotherapy; ER, estrogen receptor; HER2, human epidermal growth factor receptor 2; IDC, invasive ductal carcinoma; ILC, invasive lobular carcinoma; IORT, intraoperative radiotherapy; PR, progesterone receptor; SES, salary economic states (Based on per capita GDP data from the US Bureau of Economic Analysis in 2020, we divided the state of the patient's medical institution into high SES (≥$80000) and low SES (<$80000)).

### 3.3 Results of statistical analysis

**FIGURE 3:**
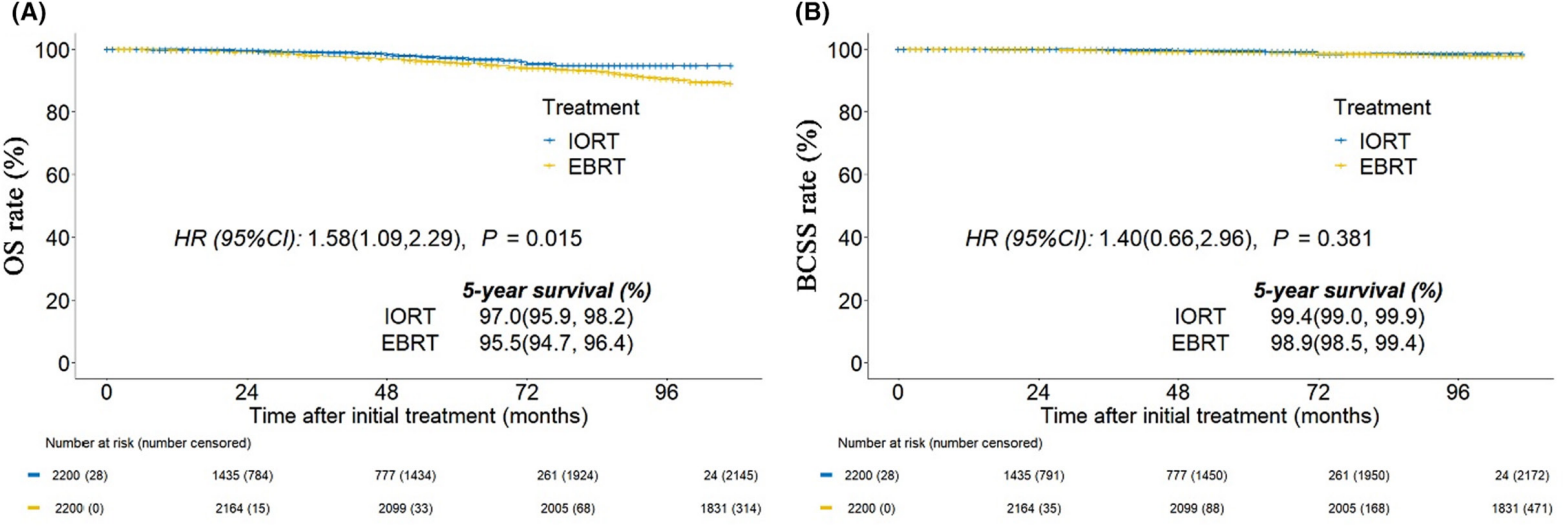
Kaplan–Meier overall survival curve of patients who received IORT or EBRT after PSM (A), and breast cancer specific survival curve after PSM (B). IORT, intraoperative radiotherapy; EBRT, external beam radiotherapy; PSM, propensity score matching; OS, overall survival; BCSS, breast cancer specific survival; Hazard ratios (HR) were used to analyze 5‐year survival. *p* ≤ 0.05 indicates statistical significance.

The median follow‐up time was 6.25 years (0–8.92 years). After the Kaplan–Meier survival analysis, the EBRT group had a worse prognosis than the IORT group in survival rate. At 5‐year survival rate, patients treated with EBRT had significantly inferior OS than IORT (95.5% vs. 97.0%, HR = 1.58, *p*= 0.015), but there was no statistical difference in BCSS (98.9% vs. 99.4%, HR = 1.40, *p* = 0.381) (Figure 3).

In multivariate COX regression analysis (Table 2), age >55 (*p* < 0.001), T2 (*p* < 0.001), and marital status (separated/divorced/widowed/unknown, *p* < 0.001) were the risk prognostic factors of OS, while PR positive (*p* = 0.027) and histological type (mixed, *p* = 0.004) seem to be associated with a better prognosis. Slightly different from this was that T2 (*p* < 0.001), N1 (*p* = 0.049), PR negative (*p* = 0.012), histological grade II (*p* = 0.015) and histological grade III (*p* = 0.011) were the risk prognostic factors of BCSS. Overall, elevated TNM stage and increased type of pathology portend a poor prognosis. It was worth noting that there was a significant difference in OS between IORT and EBRT, but not in BCSS (OS: *p* = 0.020; BCSS: *p* = 0.190).

**TABLE 2:** Multivariate Cox regression survival analysis was performed to identify independent prognostic factors for OS and BCSS in patients with BCS who underwent EBRT or IORT.

Characteristic	OS	*p*‐value	BCSS	*p*‐value
	HR (95% CI)		HR (95% CI)	
Age				
≤55	Reference		‐	‐
>55	3.43 (2.02, 5.81)	<0.001	‐	‐
T stage				
T1	Reference		Reference	
T2	2.38 (1.75, 3.22)	<0.001	3.66 (2.08, 6.46)	<0.001
N stage				
N0	Reference		Reference	
N1	1.33 (0.82, 2.16)	0.250	2.18 (1.00, 4.76)	0.049
Systemic/surgery sequence				
Neoadjuvant therapy	Reference		Reference	
Adjuvant therapy	0.74 (0.23, 2.34)	0.603	0.51 (0.07, 3.86)	0.515
No systemic therapy	1.51 (0.47, 4.83)	0.484	1.24 (0.16, 9.87)	0.841
Others	0.87 (0.17, 4.34)	0.862	0.80 (0.05, 13.30)	0.874
Chemotherapy				
No	‐	‐	Reference	
Yes	‐	‐	1.15 (0.50, 2.64)	0.747
Breast cancer type				
Non‐triple negative	Reference		Reference	
Triple negative	0.65 (0.25, 1.68)	0.374	0.70 (0.14, 3.61)	0.674
ER				
Negative	Reference		Reference	
Positive	0.65 (0.28, 1.51)	0.315	0.79 (0.17, 3.61)	0.765
PR				
Negative	Reference		Reference	
Positive	0.66 (0.45, 0.95)	0.027	0.41 (0.20, 0.82)	0.012
Marital status				
Married/unmarried or domestic partner	Reference		‐	‐
Single (never married)	1.07 (0.69, 1.64)	0.768	‐	‐
Separated/divorced/widowed/unknown	2.02 (1.56, 2.63)	<0.001	‐	‐
Histological grade				
Grade I	Reference		Reference	
Grade II	1.16 (0.87, 1.54)	0.321	3.33 (1.27, 8.75)	0.015
Grade III	1.42 (0.95, 2.12)	0.088	4.23 (1.38, 12.96)	0.011
Histological type				
IDC	Reference		‐	‐
ILC	0.81 (0.45, 1.46)	0.481	‐	‐
Mixed	0.44 (0.25, 0.76)	0.004	‐	‐
Treatment				
IORT	Reference		Reference	
EBRT	1.56 (1.07, 2.26)	0.020	1.65 (0.78, 3.49)	0.190

*Note*: *p* ≤ 0.05 indicates statistical significance.

Abbreviations: BCSS, breast cancer specific survival; CI, confidence intervals; EBRT, external beam radiotherapy; ER, estrogen receptor; HR, hazard ratios; IORT, intraoperative radiotherapy; OS, overall survival; PR, progesterone receptor.

**FIGURE 4:**
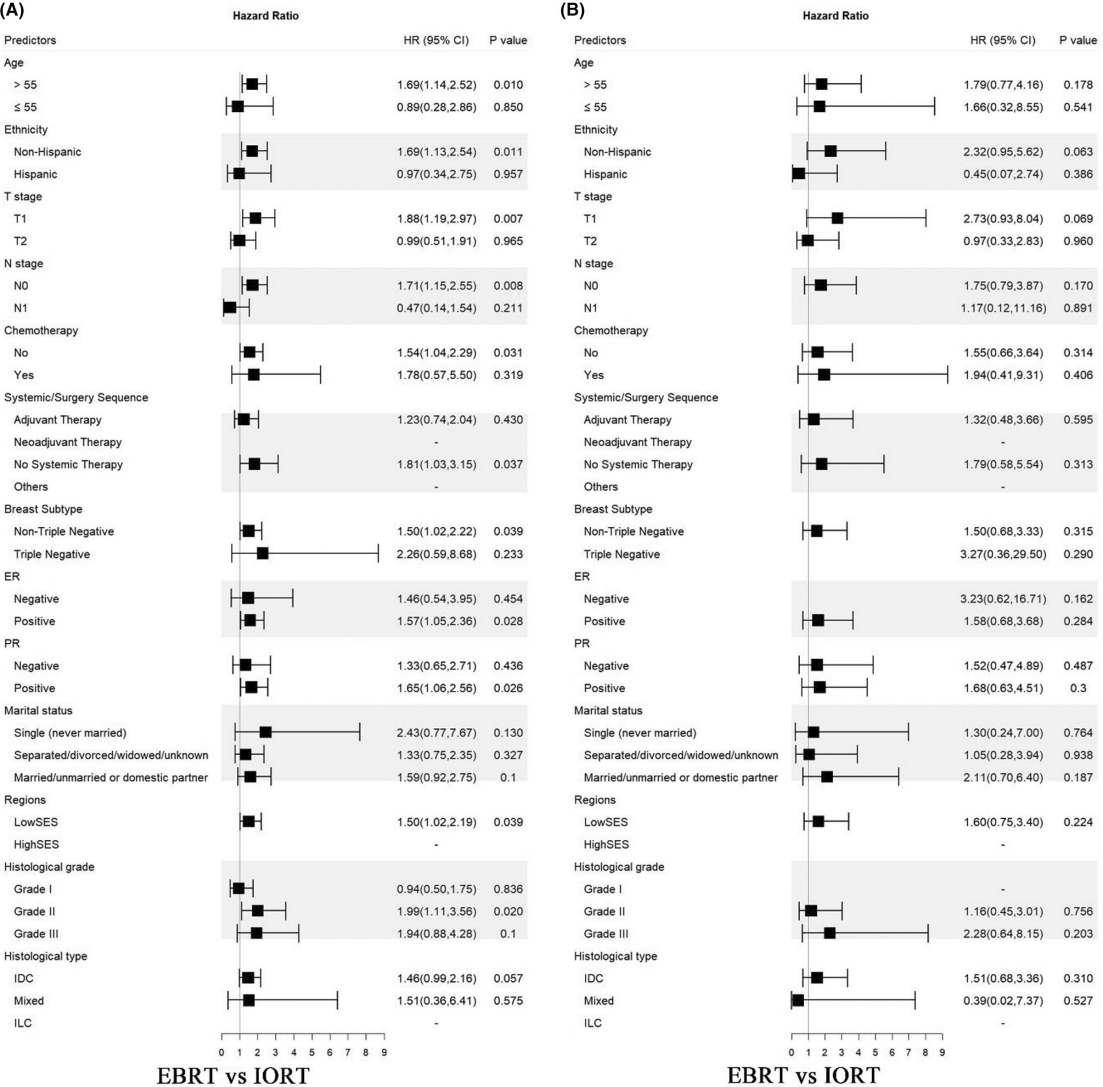
Subgroup analysis of overall survival (A) and breast cancer specific survival (B) between IORT and EBRT after PSM. HR, hazard ratios; CI, confidence intervals; ER, estrogen receptor; PR, progesterone receptor; SES, salary economic states; IDC, invasive ductal carcinoma; ILC, invasive lobular carcinoma; IORT, intraoperative radiotherapy; EBRT, external beam radiotherapy; PSM, propensity score matching. *p* ≤ 0.05 indicates statistical significance.

Further stratification of data prompted (Figure 4): with OS as an endpoint, patients age >55 years (HR = 1.69, *p* = 0.010), non‐Hispanic (HR = 1.69, *p* = 0.011), T1 (HR = 1.88, *p* = 0.007), N0 (HR = 1.71, *p* = 0.008), no chemotherapy (HR = 1.54, *p* = 0.031), no systemic therapy (HR = 1.81, *p* = 0.037), non‐triple negative breast cancer (HR = 1.50, *p* = 0.039), ER positive (HR = 1.57, *p* = 0.028), PR positive (HR = 1.65, *p* = 0.026), histological grade II (HR = 1.99, *p* = 0.020) were more likely to benefit from IORT treatment. Frustratingly, these benefits were not reflected in the analyses that used the BCSS as an endpoint.

## 4 DISCUSSION

In recent years, clinicians have been in the pursuit of the least treatment to achieve the best outcome. Relevant studies have shown that it is safe and feasible to de‐escalate radiotherapy for post‐BCS patients with a low‐risk of local recurrence.^17^, ^18^, ^19^ The formation of an ideal adjuvant radiotherapy strategy for patients should be based on risk stratification of patients. IORT is a relatively convenient form of radiotherapy, and studies have shown that IORT as an adjuvant radiotherapy is not inferior to EBRT in the overall survival of early and low‐risk breast cancer patients.^13^, ^14^ However, IORT has only been used in high selection breast cancer patients, the guidelines do not recommend it as a conventional treatment strategy used in patients who had accepted BCS.^20^, ^21^, ^22^, ^23^, ^24^ This study aimed to evaluate the application of IORT in the real world: 1. The efficacy of IORT as adjuvant radiotherapy compared with EBRT in the real world for early‐stage low‐risk post‐BCS patients; 2. To explore the most suitable candidates for IORT as adjuvant therapy. At a median follow‐up of 6.25 years (0–8.92 years) in the SEER database, the 5‐year survival rate was 97.0% with BCS‐combined IORT patients versus 95.5% with BCS‐combined EBRT patients after 1:1 PSM (*p* = 0.015), it seems that patients in IORT group had better overall survival. However, BCSS was 99.4% with BCS‐combined IORT patients versus 98.9% with BCS‐combined EBRT in 5 years (*p* = 0.381). It indicated that BCS‐combined with IORT was not inferior to that of EBRT in terms of risk of death related to breast cancer in our selected patients, which is consistent with the results of two previous large studies, ELIOT and TARGIT‐A.^13^, ^14^

We included the IORT group with the largest number of patients (*n* = 2200) in our study. It was not difficult to find that the number of patients who received IORT as adjuvant therapy had increased from 2010 to 2014 (0.25%–1.90%) but remained basically stable from 2014 to 2018 (1.90%–1.98%). A global survey of IORT applications found that IORT had been used in 35 countries and at least 44,752 breast cancer patients had received IORT.^25^ In an Italian survey of clinical practice questions, 34% used IORT as the preferred method of partial breast irradiation. Strict criteria had been established for the practice of IORT in Germany, such as age >50 years, T1N0M0, and IDC. It is estimated that about 10%–15% of patients undergoing BCS would be eligible for IORT as solo radiotherapy.^26^ It shows that IORT has been raised more attention and applied to some extent in the real world.

IORT has certain advantages over EBRT in the localization of target lesions. It must be mentioned the concept of “geographical miss”, which was first proposed by Felix Sedlmayer.^27^ It is referring to: even if titanium clips are used to locate the tumor bed during the operation, we may miss the real tumor bed due to the traction and displacement of the tissue or the high fluidity of the breast due to the rich fat content after the operation, which may be one of the reasons for the local recurrence of BCS after radiotherapy. IORT is performed immediately during surgery, which can directly irradiate the tissues around the tumor, avoid the loss of tumor bed, and improve the targeting and accuracy of radiotherapy. There is another, more obvious advantage of IORT is the direct irradiation of peritumoral tissue, avoiding unnecessary or potentially destructive radiation damage to the surrounding normal tissues and adjacent organs such as heart, lung, muscle, skin, and chest wall.^28^, ^29^, ^30^, ^31^ In addition, IORT only takes a very short radiation time, generally less than 30 min, because it is a single dose of radiation performed during the surgery. Finally, compared with EBRT, the treatment cost of IORT has been greatly reduced. It reported that IORT can save 1800 pounds per patient only in treatment, not to mention the transportation costs for treatment. Similarly, in terms of British finance, IORT can even save 13 million pounds per year.^32^ According to our patients, the savings can be as much as $7000.

By analyzing 11 randomized trials, Lis Victoria Ravani et al.^33^ found that patients treated with IORT had a higher rate of ipsilateral breast tumor recurrence (IBTR) than those treated with partial breast irradiation (PBI) and standard or moderately hypofractionated EBRT, compared with no difference in OS or disease‐free survival (DFS). Marina Guenzi et al.^34^ also found a higher local recurrence of IORT (3.4%) than hypofractionated EBRT (0.42%, 39 Gy/13 fractions, plus an individualized concomitant boost to the tumor bed up to 42–43 Gy) through 6 years of long‐term follow‐up, a finding that may be relevant to certain subgroups of patients who, based on the biology of their breast cancer, may be less amenable to IORT. Interestingly, there was no significant difference in health‐related quality of life between the regimens of IORT (1 × 23.3 Gy), PBI (10 × 3.85 Gy), hypofractionated EBRT (16 × 2.67 Gy), hypofractionated EBRT + boost (21–26 × 2.67 Gy), and simultaneous EBRT + boost (28 × 2.3 Gy).^35^

Previous studies have found that as many as 15%–30% of early breast cancer patients who undergo BCS do not receive postoperative radiotherapy.^5^ The main reasons include that some patients live far from the radiotherapy center and is not convenient to receive radiotherapy, or some patients have other comorbidities, or due to financial pressure, especially in the elderly population. Even some patients meet the surgical criteria for BCS who choose mastectomy just to avoid radiotherapy.^36^ Therefore, IORT perhaps offers a relatively good option for these pathologically low‐risk patients if they have difficulty receiving conventional radiotherapy. This is also why more and more patients prefer IORT as a post‐BCS adjutant therapy. In the Italian study,^26^ many patients preferred to choose intraoperative radiotherapy at the risk of recurrence. Otherwise, for most patients, the benefits of radiotherapy after BCS far outweigh the risks, especially for those elderly women with systemic diseases always have short life expectancy.^37^, ^38^, ^39^

With any treatment, we have to balance it with the adverse effects it brings. EBRT as a standard adjuvant treatment after BCS also brings many ineligible side effects and may achieve limited benefits in certain very low‐recurrence risk patients. Grit Welzel et al reported the IORT group had less postoperative pain in the whole body, arm, and breast, and better functional activity (*p* < 0.01) compared to EBRT.^11^ According to TARGIT‐A,^40^ grade III‐IV radiation‐related complications such as dermatitis, telangiectasis, and pain at the radiation site were significantly lower in the IORT group than in the EBRT group 20 (median follow‐up: 4 years, 0.5% vs. 2.1%, *p* = 0.002). Chronic toxicities such as skin fibrosis and telangiectasis were also lower in the IORT group than that in the EBRT group (5.9% vs. 18.1%, 0 vs. 17.7%).^12^ IORT also can achieve a better cosmetic effect because of little radiation damage to the local skin that causes an inflammatory response and fibrosis in the breast skin. Breast esthetics is a very important outcome of patients' concerns after BCS. Mohammed et al^41^ evaluated that IORT had better cosmetic results (such as breast symmetry, scar, and color change) than EBRT at either 1 or 2 years after BCS (1 year: OR 2.07, 95% CI 1.12–3.85, *p* = 0.021; 2‐year: OR 2.11, 95% CI 1.0–4.45, *p* = 0.05). Therefore, IORT has great advantages in terms of adverse effects.

As for 5‐year OS, patients who received targeted IORT represented better outcomes than those who received whole‐breast EBRT, however, the main difference came from non‐BCSS related deaths. Breast cancer mortality was much the same between groups reconfirming the non‐inferiority of targeted IORT for early breast cancer better in our study. Our results are consistent with that of ELIOT and TARGIT‐A, which may be related to the relatively lower recurrence risk of patients in the IORT group in our study (T1: 87.18%, N0: 95.64%, non‐triple‐negative breast cancer: 97.05%). TARGIT‐A adopts a risk‐adapted design,^14^ for patients will be given additional EBRT after IORT if the final pathology report shows unpredicted prespecified adverse features, which indicates that IORT should be individualized and it is necessary to carefully select patients with low‐risk of recurrence. ELIOT^13^ showed that although there was no significant difference in OS between IORT and EBRT (median follow‐up 13.1 years, *p* = 0.85), the local recurrence rate of IORT alone was higher than that of EBRT (median follow‐up 12.4 years, IORT 11% vs. EBRT 2%, *p* < 0.0001). This may be related to the relatively high lymph node involvement in ELIOT patients (T2: 13%, N positive: 26%, triple‐negative breast cancer: 7%), and it may not be sufficient to give local tumor bed radiotherapy alone in these node‐metastasis patients. For very low‐risk breast cancer (T<1 cm, histologic grade I, luminal A, and Ki‐67<14%) in the ELIOT trial, IORT was associated with a similar 10‐year local recurrence rate compared with EBRT (1.3% vs. 1.5%, *p* = 0.45), suggesting that IORT can be safely performed in highly selected low‐risk patients. In addition to this, it has also been shown that in women aged 65 years and older with low‐risk, lymph node‐negative, hormone receptor‐positive, early‐stage primary breast cancer, the absence of radiotherapy is associated with an increased incidence of local recurrence, but has no detrimental effect on distant recurrence as a first event or on overall survival.^42^ In our further subgroup analysis, IORT was more suitable for patients >55 years old, with T1, N0, ER positive, PR positive, non‐triple negative breast cancer, and grade I‐II histology, also consistent with the patients enrolled in the TARGIT‐A study. Therefore, we believe that EBRT after BCS is still a routine treatment for breast cancer patients, but IORT is also a viable alternative to EBRT for certain patients with low‐risk breast cancer.

This study has the following limitations. First, it could not address detailed information on local recurrence and distant metastasis. Local recurrence is an important factor in evaluating local control of radiotherapy. Second, the SEER database can't provide specific information on radiation dose, endocrine therapy, anti‐HER2 therapy, or chemotherapeutic, which may cause potential bias in our study.

## 5 CONCLUSION

The results showed that patients who received IORT had better 5‐year OS than those who received EBRT, but there was no significant difference between the two groups in terms of 5‐year BCSS. Those older than 55 years, T1, N0, non‐triple negative breast cancer, ER positive, PR positive, and grade II histology are more likely to benefit from IORT treatment. Therefore, IORT may be a reasonable alternative to EBRT for highly selective breast‐conserving patients with early‐stage breast cancer.

## AUTHOR CONTRIBUTIONS

**Dexun Sun:** Conceptualization (lead); data curation (equal); formal analysis (equal); validation (equal); visualization (equal); writing – original draft (lead). **Guanhua Lu:** Methodology (equal); validation (equal); visualization (equal); writing – original draft (lead); writing – review and editing (equal). **Fenmei Liang:** Data curation (supporting); formal analysis (supporting); investigation (supporting). **Wangjian Zhang:** Methodology (equal); software (supporting); supervision (supporting); visualization (supporting). **Tao Zeng:** Formal analysis (supporting); methodology (supporting); validation (supporting). **Yun Ling:** Investigation (supporting); methodology (supporting); validation (supporting). **Haojie Peng:** Investigation (supporting); methodology (supporting); visualization (supporting). **Ting Xia:** Funding acquisition (supporting); investigation (supporting); supervision (supporting); validation (supporting). **Meilin Hu:** Data curation (supporting); formal analysis (supporting); software (supporting). **Xinxin Chen:** Conceptualization (equal); funding acquisition (lead); project administration (lead); supervision (lead); writing – review and editing (equal).

## FUNDING INFORMATION

This study was funded by Guangzhou Basic Research Programme Municipal School (Hospital) Joint Funded Foundation and Application Basic Research Project (202201020115).

## CONFLICT OF INTEREST STATEMENT

The authors declare that they have no competing interests.

## ETHICS STATEMENT

All practices used in the research involving human subjects complied with the 1964 Helsinki Declaration and any later revisions or equivalent ethical standards, as well as with institutional and/or national research committee ethical standards.

## CONSENT

The SEER database is publicly available and retrospective, and therefore informed consent is not applicable.

## Data Availability

Data sharing is not applicable to this article as no new data were created or analyzed in this study.
